# Sulfated polysaccharide-coated protein nanoparticles for enhanced delivery of psoralen in breast cancer therapy

**DOI:** 10.3389/fphar.2026.1888270

**Published:** 2026-10-20

**Authors:** Huiqiong Guo, Jing Liang, Haili Huang, Zimo Zhang, Xin Huang, Zongxi Sun, Yinghong Huang

**Affiliations:** 1 Zhuang Medicine Clinical Medical College, Guangxi University of Chinese Medicine, Nanning, China; 2 Second Clinical Medical School, Guangxi University of Chinese Medicine, Nanning, China; 3 School of Chinese Materia Medica, Beijing University of Chinese Medicine, Beijing, China

**Keywords:** antitumor activity, breast cancer therapy, fucoidan, nano-drug delivery system, psoralen, zein nanoparticles

## Abstract

**Introduction:**

Psoralen (PSO) is an anticancer agent with a potential role in breast cancer management. Its clinical application, however, is significantly limited by poor aqueous solubility and low bioavailability. Therefore, the development of advanced PSO delivery systems is essential to enhance its therapeutic potential.

**Methods:**

In this study, a novel nano-delivery platform was constructed by encapsulating PSO into zein nanoparticles (NPs) followed by surface modification with fucoidan (Fu), yielding PSO-Z/F NPs.

**Results:**

Successful surface modification was confirmed by a reversal in ζ potential from positive values to −24.8 ± 0.02 mV, an increase in particle size to 129.23 ± 0.18 nm, characteristic changes in sulfate-related Fourier transform infrared spectroscopy (FTIR) bands, and the appearance of an S 2p signal in X-ray photoelectron spectroscopy (XPS). The PSO-Z/F NPs exhibited a uniform spherical morphology, with an encapsulation efficiency of 73.13 ± 0.98% and a drug-loading capacity of 5.40 ± 0.35%. Cellular uptake of PSO-Z/F NPs was 1.6-fold higher than that of uncoated PSO-Z NPs (*P* < 0.05). *In vitro*, PSO-Z/F NPs significantly suppressed the proliferation, migration, and invasion of 4T1 breast cancer cells. Specifically, wound closure rate decreased from 29.13 ± 3.33 % in the control group to 5.02 ± 2.86% (*P* < 0.001). Compared with the PSO group, the number of migrated cells decreased from 220 ± 33 to 146 ± 20 (*P* < 0.01), while that of invaded cells decreased from 144 ± 21 to 90 ± 8 (*P* < 0.05). Moreover, PSO-Z/F NPs increased the total apoptosis rate from 6.45% in the control group to 17.98% (*P* < 0.01). In addition, hemolysis assays showed reduced hemolytic activity, while serum-incubation studies confirmed improved colloidal stability, as evidenced by minimal changes in particle size and PDI over 7 days. Notably, *in vivo* and *ex vivo* fluorescence analysis of tumors and major organs in 4T1 tumor-bearing mice showed efficient tumor accumulation of PSO-Z/F NPs, and pharmacodynamic studies indicated enhanced tumor growth inhibition with an acceptable biosafety profile.

**Discussion:**

Overall, PSO-Z/F NPs may provide a potential nanoparticle-based delivery strategy for improving the therapeutic application of psoralen in breast cancer.

## 1 Introduction

Breast cancer is now the most common cancer in women, surpassing lung cancer in incidence (Bray et al., 2024; Siegel et al., 2025). Owing to advances in precision medicine, the management of breast cancer has become increasingly multidisciplinary and individualized, encompassing surgery, radiotherapy, chemotherapy, endocrine therapy, targeted therapy, and immunotherapy (Barzaman et al., 2020; Katsura et al., 2022). Despite these therapeutic advances, substantial clinical challenges remain. For hormone receptor-positive breast cancer, the emergence of intrinsic or acquired resistance to endocrine therapy may compromise treatment efficacy and contribute to disease progression, particularly in advanced-stage patients. In addition, triple-negative breast cancer lacks well-defined therapeutic targets and is associated with limited treatment options, aggressive biological behavior, and a high risk of recurrence and metastasis (Asleh et al., 2022; Nolan et al., 2023). Therefore, the pressing challenge lies in identifying efficacious drugs to combat drug resistance, metastasis and recurrence of breast cancer.

Psoralen (PSO) is a natural furanocoumarin compound with anti-inflammatory, immunomodulatory, anti-tumor and drug resistance reversal properties (Galiatsatos et al., 2024; Tripathi et al., 2023). PSO may represent a promising therapeutic option for breast cancer, given its reported antitumor activity. Its antitumor effects have been associated with the induction of cell cycle arrest and cell death, as well as the inhibition of metastasis and angiogenesis (Ren et al., 2020). In breast cancer studies, PSO plays several roles. It triggers apoptosis in breast cancer cells by activating the expression of phosphorylated IRAK1 and NF-κB (Liu et al., 2021), and altering the expression of β-catenin leading to apoptosis in various breast cancer cells (Wang et al., 2018). Furthermore, PSO impedes the invasion and metastases (Wu et al., 2013; Zhang et al., 2018) of breast cancer cells by decreasing the expression of matrix metalloproteinases (MMP-2, MMP-9) and inhibiting epithelial-mesenchymal transition (EMT) process. PSO also has synergistic with Chemotherapeutics, increasing the toxicity towards tumor cells, and reverse multidrug resistance in breast cancer cells (Huang et al., 2018; Jiang et al., 2016).

However, PSO exhibits poor water solubility and low bioavailability, which hinders its clinical applications. The advancement of novel drug delivery systems represents a significant solution to these challenges (Bhandari et al., 2025; Cao et al., 2024), and markedly strengthens the activity of PSO in cancer therapy (Du et al., 2019; Li et al., 2021). Zein and fucoidan are natural polymers characterized by high biocompatibility and biodegradability and they are effective hydrophobic drug delivery nanocarriers (Chen et al., 2024). Zein, a plant-based protein derived from maize (Zea mays L.), serves as an affordable and readily available polymer that exhibits gastro-resistant and mucoadhesive characteristics, enabling the encapsulation of bioactive compounds possessing hydrophilic, hydrophobic, and amphiphilic traits (Sudhakar Gomte et al., 2025). Zein has significant advantages in the construction of controlled-release carriers (Oleandro et al., 2024). It can solidify spontaneously without chemical modification, avoiding the use of toxic chemical crosslinkers, thus exhibiting good biosafety and application prospects (Wen et al., 2022). Nanocarriers based on zein efficiently encapsulate various bioactive compounds (André de Almeida Campos et al., 2023), and exhibit a strong affinity for passive tumor targeting *via* the permeable tumor vasculature, leading to the accumulation of the encapsulated drug in the tumor tissue (Elzoghby et al., 2017).

Fucoidan (Fu), a marine-sulfated polysaccharide with multiple targets, can be utilized to produce nanoparticles with diverse therapeutic uses (Chung et al., 2020; Ettoumi et al., 2023; Venkatesan et al., 2022). In the tumor microenvironment (TME), Fu selectively binds to L-selectin, Toll-like receptors (Oliveira et al., 2020), and can interacts with overexpressed P-selectin receptors (Ho et al., 2023). This biopolymer shows promising potential for cancer treatment due to its efficacy, safety, and targeted activity (Etman et al., 2021; Oliveira et al., 2021).

Recent studies demonstrated that Zein-Fucoidan nanosystem shows pronounced uptake by tumor cells and significant cytotoxicity (Moustafa et al., 2023). The coating with fucoidan increased encapsulation efficiency and stabilization capacity (Lu et al., 2024), while markedly enhancing specific binding affinity for metastatic tumor cells and simultaneously inhibiting tumor invasiveness (Lin et al., 2025; Tsai et al., 2025). This nanosystem show promise as a targeted delivery system for water-insoluble drugs.

Herein, PSO was encapsulated within Zein-NPs by antisolvent precipitation. Following this, Fu adhered to the PSO-Zein NPs through electrostatic forces to create a new drug delivery platform, termed PSO-Z/F NPs (Scheme 1). The formulation was designed to improve the delivery performance and antitumor potential of PSO in breast cancer. In this study, PSO-Z/F NPs exhibited enhanced cellular uptake and prolonged drug retention in tumor tissues compared to the relevant control formulations. Moreover, PSO-Z/F NPs demonstrated improved inhibitory effects on tumor progression in both *in vitro* and *in vivo* models. Qualitative histological examinations of major organs did not reveal any significant treatment-related pathological abnormalities under the tested conditions, indicating preliminary *in vivo* biocompatibility. Overall, PSO-Z/F NPs may address certain delivery-related limitations of PSO and offer a potentially effective nanocarrier-based strategy for breast cancer treatment. Further research is necessary to explore their biodistribution, long-term safety, targeting performance, and translational potential.

**SCHEME 1:**
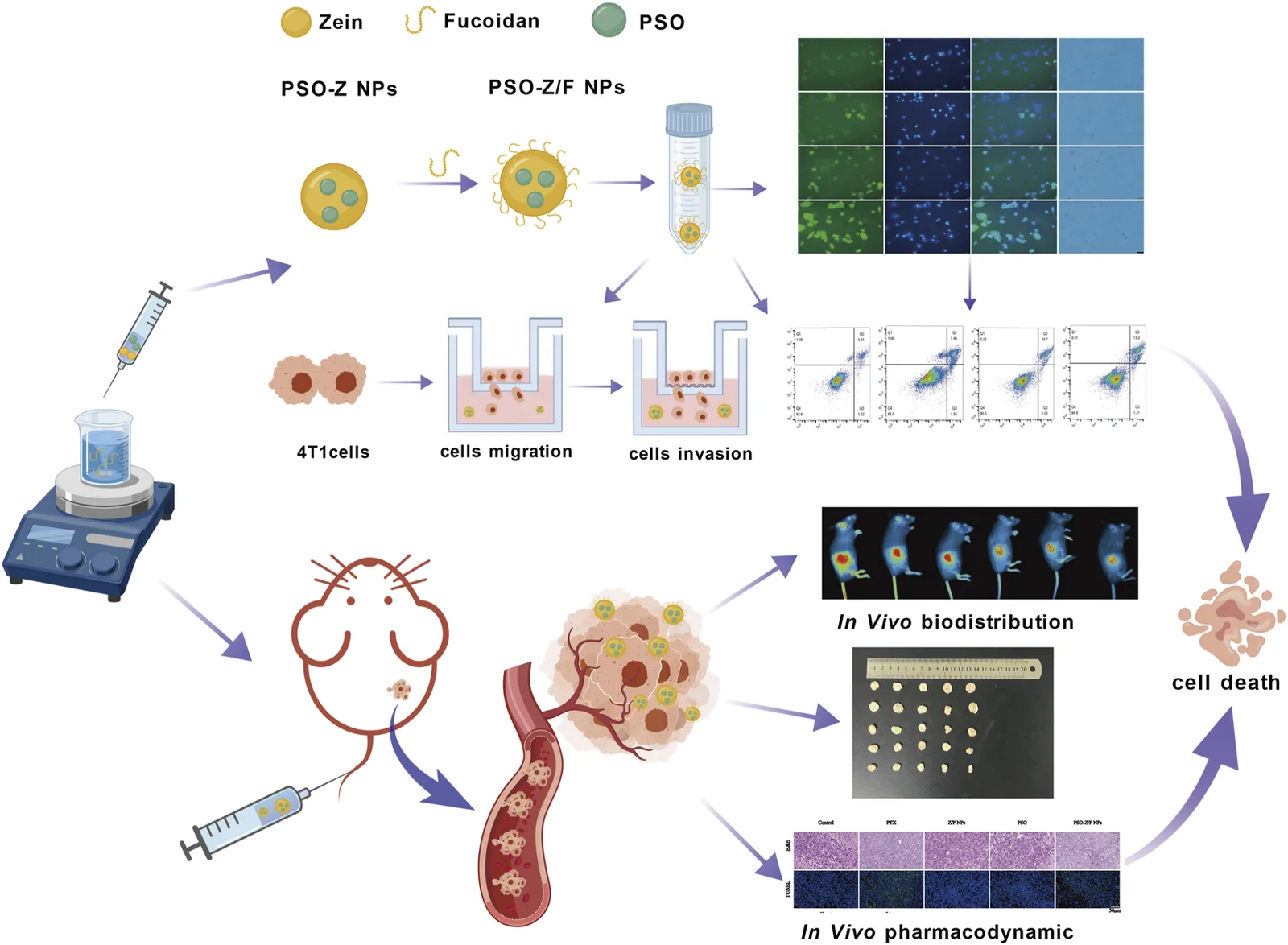
Diagram illustrating the “sulfated polysaccharide (Fucoidan)-coated protein (Zein) nanoparticles for enhanced delivery of psoralen in breast cancer therapy”.

## 2 Materials and methods

### 2.1 Reagents

PSO (98% purity) was purchased from Dalian Meilun Biotech Co., Ltd. China. Zein and Fucoidan (from Laminaria japonica, 40–100 kDa molecular weight, 25% sulfate content) were provided by Shanghai Yuanye BioTechnologyCo., Ltd. China. The Annexin-FITC Apoptosis Detection Kit and Matrix-Gel™ Matrix Gel were obtained from Beyotime (Shanghai, China). penicillin, and streptomycin were purchased from Solarbio (Beijing, China). RPMI-1640 cell culture medium, trypsin EDTA were purchased from Thermo Scientific (Gibco, United States). Cy5.5, Dimethyl Sulfoxide (DMSO) and fluorescein isothiocyanate (FITC) were bought from Aladdin Reagents (Shanghai, China). The murine breast cancer cell line 4T1 and FBS were obtained from Wuhan Pricella Biotechnology Co., Ltd. China. CCK-8 Assay Kit was bought from Beijing Lanjie Ke Technology Co., Ltd. China.

### 2.2 Fabrication of PSO-Z/F NPs

The PSO-Z/F NPs were synthesized *via* the antisolvent precipitation method (Yu et al., 2022; Yu et al., 2020). Initially, 750 µL of zein (70 mg/mL dissolved in DMSO) and 250 µL of PSO (20 mg/mL dissolved in DMSO) were mixed thoroughly to form a homogeneous organic phase. Then, the organic phase was added dropwise into 10 mL Fu aqueous solution (1 mg/mL) at a rate of approximately 10 s per drop (equivalent to approximately 0.02 mL/min) under rapid magnetic stirring at 1,000 rpm. Upon completion of the addition, stirring ceased to yield the PSO-Z/F NPs. Subsequently, the nanoparticles underwent centrifugation at 10,000 rpm for 10 min to eliminate excess drugs. Combinations of PSO-Z NPs and Z/F NPs were also prepared following a similar procedure, with the exception that the Z/F NPs were synthesized without the inclusion of PSO and the PSO-Z NPs were synthesized without the inclusion of Fu. The resulting nanoparticle dispersion was divided into two portions: one portion was freeze-dried for physicochemical characterization, while the other portion was used directly for biological experiments.

### 2.3 Characterization

#### 2.3.1 Physicochemical characterization

The hydrodynamic diameter, polydispersity index (PDI) and ζ potential were determined with a ZS90 Particle Size Analyzer from Malvern Instruments. Nanoparticle morphology was investigated by transmission electron microscopy (TEM, Hitachi HT7700, Japan). Chemical functional groups were analyzed using Fourier transform infrared spectroscopy (FTIR, Thermo Scientific NIR-Antaris II, United States). Crystalline structure of the nanoparticles was characterized using an X-ray diffractometer (XRD, UltimalV, Japan) and Differential scanning calorimetry (DSC, Q20, United States). X-ray diffractometer equipped with a nickel filter and CuKα radiation (λ = 0.154 nm) at 40 kV and 40 mA. The temperature of Differential scanning calorimetry was elevated from 25 °C to 250 °C at a rate of 10 °C/min under a nitrogen purge of 50 mL/min. Secondary structure analysis was performed using a Circular Dichroism (CD, Chirascan™, United Kingdom). Far-UV CD spectra were recorded from 180 to 350 nm at room temperature, using a scan rate of 100 nm/min. The surface elements analysis was identified using an X-ray photoelectron spectroscopy (XPS, Thermo SCIENTIFIC Nexsa, United States).

#### 2.3.2 HPLC determination of PSO

PSO was quantified by an HPLC method following previous literature (Huang et al., 2018; Liu et al., 2021; Yuan et al., 2019). Chromatographic separation was performed on a Gemini C18 column (250 mm × 4.6 mm, 5 μm). The mobile phase consisted of acetonitrile and water (55:45, v/v) and was delivered at a flow rate of 1.0 mL/min under isocratic elution. The injection volume was 20 μL, and the detection wavelength was set at 245 nm. The column temperature was maintained at 25 °C.

Although a different HPLC instrument (Agilent, 1260infinity, US) was used in the present study, the key chromatographic conditions were unchanged from those of the previously validated method. The applicability of the method on the current instrument and in the zein nanoparticle matrix was confirmed by a PSO calibration curve and chromatographic specificity assessment using blank Z/F NPs, PSO standard solution, and PSO-Z/F NPs.

#### 2.3.3 Encapsulation efficiency and loading capacity

The total and free PSO contents of PSO-Z/F NPs were measured by HPLC with detection at 245 nm, using the method described above. To determine total PSO, a freshly prepared PSO-Z/F NPs suspension (1 mL) was mixed with 4 mL methanol and sonicated for 10 min to disrupt the nanoparticles and release PSO. The resulting methanolic solution was filtered through a 0.22 μm organic membrane filter prior to HPLC analysis. To quantify free PSO, equal volumes of freshly prepared PSO-Z/F NPs suspension were placed into 10 kDa ultrafiltration tube (Millipore Amicon Ultra) and centrifuged at 12,000 rpm for 15 min at 25 °C. The filtrate was collected and subjected to a constant volume of methanol and a 0.22 μm organic filter membrane. The free PSO content was determined using HPLC. The encapsulation efficiency (EE%) and drug loading (DL%) were calculated according to the following equations:

$$\text{EE }\left(\%\right)=\left(W_{total\;PSO}-W_{free\;PSO}\right)\;/\;W_{total\;PSO}\;x100$$

$$\text{DL }\left(\%\right)=\left(W_{\text{total}\;\text{PSO}}-W_{\text{free}\;\text{PSO}}\right)\;/\;\left(W_{\text{PSO},\text{initial}}+W_{\text{zein},\text{initial}}+W_{\text{Fu},\text{initial}}\right)\times 100$$

#### 2.3.4 *In vitro* drug release investigation

The *in vitro* release profiles were performed using the dialysis bag diffusion technique. Briefly, 10 mL of PSO-Z/F NPs dispersion, containing 2.1 mg of PSO, was sealed in dialysis bags (MWCO: 8,000–14,000 Da). The dialysis bag was immersed in 90 mL of phosphate buffer solution (PBS) at pH 5.5, 6.5, or 7.4 containing 0.5% (v/v) Tween 80. The release study was performed at 37 °C with continuous stirring at 100 rpm. At predetermined time intervals (1, 2, 4, 8, 12, 24, 36, and 48 h), 1 mL of release medium was sampled and replaced with an equal volume of pre-warmed fresh medium to maintain a constant volume. The collected samples were filtered through a 0.22 μm membrane filter and directly analyzed by HPLC described above for PSO quantification. The cumulative release of PSO was calculated after correction for the volume withdrawn and replaced at each sampling point. All experiments were performed in triplicate.

### 2.4 *In vitro* stability

The serum stability of Z/F NPs, PSO-Z NPs, and PSO-Z/F NPs was evaluated by incubation in 50% serum-containing medium at 37 °C and protected from light. Aliquots were collected at 0, 1, 3, 5, and 7 days. At each time point, the hydrodynamic diameter and PDI of the nanoparticles were determined using a Zetasizer (Malvern Zetasizer Nano ZS90, United Kingdom).

To assess drug leakage during storage, freshly prepared PSO-Z/F NPs were stored in PBS at 4 °C and in 10% serum-containing medium at 37 °C, respectively, with protection from light. At predetermined time intervals (0, 1, 3, 5, and 7 days), the free and total PSO contents were determined using the same method as that used for encapsulation efficiency determination. The leakage rate was calculated as follows:

$$\text{Leakage rate }\left(\%\right)=\left(W_{\text{free},t}-W_{\text{free},0}\right)\;/\;\left(W_{\text{total},0}-W_{\text{free},0}\right)\times 100$$

### 2.5 Hemolytic test

For the hemolysis assay, fresh whole blood was collected from three healthy female BALB/c mice (6–8 weeks old, 18–22 g) *via* retro-orbital bleeding. Blood samples were collected into heparinized tubes and processed immediately after collection. All animal procedures were approved by the Animal Ethics Committee of Guangxi University of Chinese Medicine (NO: DW20241,220-01). The blood samples were centrifuged at 4,000 rpm for 10 min to isolate red blood cells (RBCs). RBCs are washed repeatedly with PBS (pH 7.4) until the supernatant becomes clear and colorless. A 4% RBC suspension is then prepared in PBS. Z/F NPs, PSO-Z NPs, and PSO-Z/F NPs were evaluated at PSO-equivalent concentrations of 105 and 210 μg/mL. The concentration of 105 μg/mL corresponded to the treatment concentration utilized in the cell experiments, while 210 μg/mL represented a two-fold higher concentration for assessing high-dose hemocompatibility. Each nanoparticle suspension was mixed with a 4% RBC suspension at a volume ratio of 1:9 and incubated at 37 °C for 6 h. PBS (0.9% sodium chloride solution) and 0.1% Triton X-100 served as the negative and positive controls, respectively. Following incubation, the samples were centrifuged at 400 *g* for 5 min, and the absorbance of the supernatants was measured at 450 nm using a microplate reader. The hemolysis rate was calculated using the following equation:

$$\text{Hemolysis rate }\left(\%\right)=\left(\text{OD value of experimental group }-\text{ OD value of negative group}\right)/\left(\text{OD value of positive group }-\text{ OD value of negative group}\right)\times 100.$$

### 2.6 Cell culture

The 4T1 cells were grown in RPMI 1640 supplemented with 10% FBS, 100 units/mL penicillin, 100 μg/mL streptomycin and 37 °C in 5% CO_2_.

### 2.7 *In vitro* cytotoxicity studies

CCK-8 was used to assess the cytotoxicity of PSO-Z/F NPs. 4T1 cells (1 × 10^4^ cells per well) were seeded in 96 well plates and adhered overnight. The cells were subsequently treated with free PSO, PSO-Z NPs, PSO-Z/F NPs, and Z/F NPs for 48 h. The concentrations of free PSO, PSO-Z NPs, and PSO-Z/F NPs were normalized to an equivalent PSO concentration of 105 μg/mL. The Z/F NPs group was administered at a formulation-equivalent concentration corresponding to the amount of Z/F components present in the PSO-Z/F NPs treatment group. Untreated cells cultured in complete medium served as the control group. After 48 h of incubation, 10 μL of CCK-8 reagent was added to each well containing 100 μL of culture medium, followed by incubation for an additional 2 h. The absorbance was measured at 450 nm using a multimode microplate reader. Cell viability was expressed as a percentage relative to that of the untreated Control group. All experiments were independently performed in triplicate.

### 2.8 Cellular uptake

Qualitative detection of cell uptake was investigated using a fluorescence microscope (Olympus IX73, Japan) and quantitative analysis was carried out by a flow cytometry (FCM, BD FACSMelody, United States). The nanoparticles were labelled with FITC (2 μg/mL) and PSO (20 mg/mL) were dissolved separately in DMSO. The FITC solution was then added to the PSO solution at a volume ratio of 1:4. Subsequently, 250 μL of this FITC–PSO mixture was combined with 750 μL of Zein solution (70 mg/mL). The resulting mixture was added dropwise to a Fu solution (1 mg/mL) under continuous magnetic stirring. Afterward, the mixture was centrifuged at 10,000 rpm for 10 min to remove excess FITC. All procedures were conducted under light-protected conditions. The resulting nanoparticles were designated as PSO-FITC@Z/F NPs. PSO-FITC@Z NPs were prepared in the same way except Fu. 4T1 cells were seeded in 6-well plates at a density of 1 × 10^5^ cells per well and allowed to adhere for 24 h. The cells were then treated with PSO-FITC@Z NPs or PSO-FITC@Z/F NPs at a PSO-equivalent concentration of 105 μg/mL and incubated at 37 °C under 5% CO_2_ for 2 or 6 h. Subsequently, cells were rinsed twice with PBS and immobilized with 4% paraformaldehyde for 15 min. Finally, after staining with DAPI (10 μg/mL) for 15 min, fluorescence microscope images were captured and fluorescence signals were collected. The images were captured at 20× objective. 4T1 cells were treated with PSO-FITC@Z NPs or PSO-FITC@Z/F NPs and incubated for 2 or 6 h at 37 °C in 5% CO_2_. After incubation, the supernatant was removed and the cells were washed three times with PBS. Cells were then detached with 500 μL of EDTA-free trypsin for 15 min at 37 °C, and digestion was terminated by adding 2 mL of complete medium. Following centrifugation at 800 rpm for 5 min, the cell pellet was resuspended in 1 mL of PBS and immediately analysed by flow cytometry within 1 h. The data were analyzed using FlowJo software (version 10.10). All experiments were independently performed in triplicate.

### 2.9 Wound healing assay

Wound healing experiments investigated the effect of different treatments on 4T1 migration. 4T1 cells were seeded 5 × 10^6^ cells/well and incubated 24 h. Z/F NPs, PSO and PSO-Z/F NPs (calculated as PSO, 105 μg/mL) were introduced for 48 h uniformly wide wounds were marked vertically with a sterile gun tip, washed and photographed at 0 and 48 h. Gray values of wounds were measured using ImageJ software. The corresponding grayscale-derived wound healing rate was calculated as:

$$\text{Wound healing rate }\left(\%\right)=\left(\text{wound area at }0\;h-\text{wound area at }48\;h\right)/\text{wound area at }0\;h\times 100\%$$

### 2.10 Migration and invasion assay

Cell migration and invasion were assessed in a transwell cell culture system. In the migration assay, 4T1 cells (1 × 10^5^ cells/well) were plated in the upper chamber and exposed to the following treatments: Z/F NPs, PSO, and PSO-Z/F NPs (calculated as PSO, 105 μg/mL). After 9 h, unmigrated cells were removed, and migrated cells on the lower side were fixed with 4% paraformaldehyde, stained with 0.1% crystal violet, and observed under a microscope (CKX53, Olympus). The invasion test involved precoating the transwell membrane with 100 μL of matrixgel. Four non-overlapping fields of view were randomly selected to count the number of migratory or invasive cells. Each experiment was independently repeated at least three times.

### 2.11 Cell apoptosis experiment

Apoptosis of cells was measured by FCM after AnnexinV-FITC/PI staining. Briefly, 4T1 cells (1 × 10^5^ cells/well) were cultured in 6well plates for 24 h before treatment. After 48 h exposed to Z/F NPs, PSO, and PSO-Z/F NPs (calculated as PSO, 105 μg/mL), the cells were harvested, washed with PBS and stained with fluorescent Annexin V-FITC and propidium iodide (PI). Samples were analyzed within 1 h on FCM with 488 nm excitation. Total apoptosis was calculated as the sum of early and late apoptotic percentages.

### 2.12 Animal model generation

4T1 cells in logarithmic growth phase with viability exceeding 90% were trypsinized, collected, washed with PBS, and resuspended. The cell density was adjusted to 1 × 10^7^ cells/mL, and the resulting suspension was kept on ice until use. Eight-week-old female BALB/c mice were shaved on the right flank and the skin was disinfected. Using an insulin syringe, 100 μL of the cell suspension was injected subcutaneously into the right flank. A visible bleb formation at the injection site confirmed successful inoculation. Mice were subsequently monitored daily for general condition, activity, and tumor growth. When the size of the tumor reached 50 mm^3^, all animals were randomly assigned to different groups (n = 5 per group) using a random number table, and allocation concealment was applied during grouping and treatment to avoid selection bias. Tumor volume using the formula:

$$\text{Tumor volume}=\text{tumor length ×}{\left(\text{tumor}\;\text{width}\right)}^{2}\;/\;2$$

### 2.13 *In vivo* biodistribution

The *in vivo* imaging system Meta Morph MIIS (UVP Analytical Jena, Germany) was employed to investigate the biodistribution of NPs in mice with tumors. The nanoparticles were labeled with the near-infrared dye Cy5.5. In short, combine the Cy5.5 solution (0.5 mg/mL) with the PSO solution (20 mg/mL) in a volume ratio of 1:4. Subsequently, mix this solution with the Zein solution (70 mg/mL) in a volume ratio of 1:3. Finally, while stirring magnetically, add the resulting mixture to the Fu solution (1 mg/mL). The resulting mixture was centrifuged at 10,000 rpm for 10 min to remove excess Cy5.5, yielding PSO-Cy5.5@Z/F NPs. PSO-Cy5.5@Z NPs were prepared following the same procedure, except that no fucoidan was added. Afterwards, 100 μL of PSO-Cy5.5@Z NPs and PSO-Cy5.5@Z/F NPs were injected into 4T1 tumor-bearing mice, respectively, while the control group received PBS injection. *In vivo* fluorescence imaging was performed at scheduled time points (4, 12, 24, 48, 72, and 96 h). Following the final imaging session, all mice were euthanized, and major organs were harvested for *ex vivo* imaging. Each group consisted of three biological replicates.

### 2.14 Antitumor therapy assessment

Based on the observed tumor accumulation and retention of the Cy5.5-labeled drug-loaded nanoparticles, the antitumor efficacy of PSO-loaded nanoparticles was subsequently evaluated in a subcutaneous breast cancer syngeneic mouse model. The experimental design is presented in Figure 6A. When tumor volume reached approximately 50 mm^3^, all animals were randomly allocated to five groups (n = 5 per group) using a random number table. To minimize selection bias during group allocation and treatment, allocation concealment was employed (Tuohan et al., 2026). The animals then received intravenous injections of the following: PBS (10 mL/kg), paclitaxel (PTX; 3 mg/kg), free PSO (3 mg/kg), Z/F NPs (at a carrier-material dose equivalent to that in the drug-loaded group), and PSO-Z/F NPs (at a PSO-equivalent dose of 3 mg/kg). As shown in Figure 6A, treatments were administered once every 3 days for 14 days. Mice were monitored every other day for clinical signs. Body weight and tumor volume were recorded every other day. According to the approved protocol, humane endpoints were defined as follows: loss of ≥20% of the initial body weight; ulceration in ≥10% of the tumor area; a tumor diameter of ≥1.7 cm; or a tumor weight exceeding 10% of body weight. After the 14-day treatment period, the mice were euthanized, and tumors and major organs (heart, liver, spleen, lung, and kidney) were harvested. For histopathological assessment and terminal deoxynucleotidyl transferase dUTP nick-end labeling (TUNEL) staining, the harvested organs were processed according to standard histological protocols. Sections were stained with hematoxylin and eosin (H&E) for histopathological evaluation and subjected to TUNEL staining. All stained tissue sections were digitized using a high-resolution slide scanner and independently evaluated by two board-certified pathologists in a double-blind manner (Zheng et al., 2025).

### 2.15 Data analysis

Results are presented as mean ± standard deviation (SD) of at least three separate experiments. All statistical differences were assessed using GraphPad Prism 10.0 software. Between-group differences were assessed by one-way analysis of variance (ANOVA) followed by Tukey’s *post hoc* test for multiple comparisons. A P value of less than 0.05 was deemed statistically significant. All figures and graphs were created using Adobe Illustrator software.

## 3 Results and discussion

### 3.1 Characterization of PSO-Z/F NPs

#### 3.1.1 Analysis of PSO

The calibration curve of PSO was linear over the concentration range of 5–80 μg/mL, with the regression equation of y = 67.356 x + 187.03 and a correlation coefficient of *R* ^*2*^ = 0.9992 (Supplementary Figure S1). As shown in Supplementary Figure S2, no interfering peak was observed at the retention time of PSO in the chromatogram of blank Z/F NPs, confirming the specificity of the method in the present formulation matrix.

#### 3.1.2 Morphology, particle size, zeta potential, EE and DL

The prepared nanoparticle suspension appeared clear (Supplementary Figure S3). TEM image indicated that the nanoparticles exhibited a near-spherical morphology (Figures 1E,G). Notably, compared with PSO-Z NPs, the particle size of PSO-Z/F NPs were increased from 92.13 ± 0.74 nm to 129.23 ± 0.18 nm (Figures 1A–C). The slightly larger blank Z/F NPs (135.75 ± 0.92 nm) than PSO-Z/F NPs (129.23 ± 0.18 nm) may reflect drug-induced compaction of the zein matrix. As shown in Sections 3.1.3, 3.1.6, PSO loading disrupts α-helices and promotes β-sheet-rich aggregation *via* hydrophobic interactions and π–π stacking, forming a denser core and slightly smaller hydrodynamic diameter. Similar drug-loading-induced size decreases have been reported for zein nanoparticles (Jiao et al., 2018; Luque-Alcaraz et al., 2025; Mu et al., 2025; Yin et al., 2025). At the same time, the ζ potential reversed from positive to negative, which is −24.8 ± 0.02 mV (Figure 1D). The sulfate and carboxyl groups on fucoidan contributed to the negative charge, demonstrating its effective attachment (Tsai et al., 2025). As shown in Table 1, the encapsulation efficiency (EE) of PSO-Z/F NPs was (73.13 ± 0.98) %, while the average drug loading (DL) was (5.40 ± 0.35) %.

**FIGURE 1:**
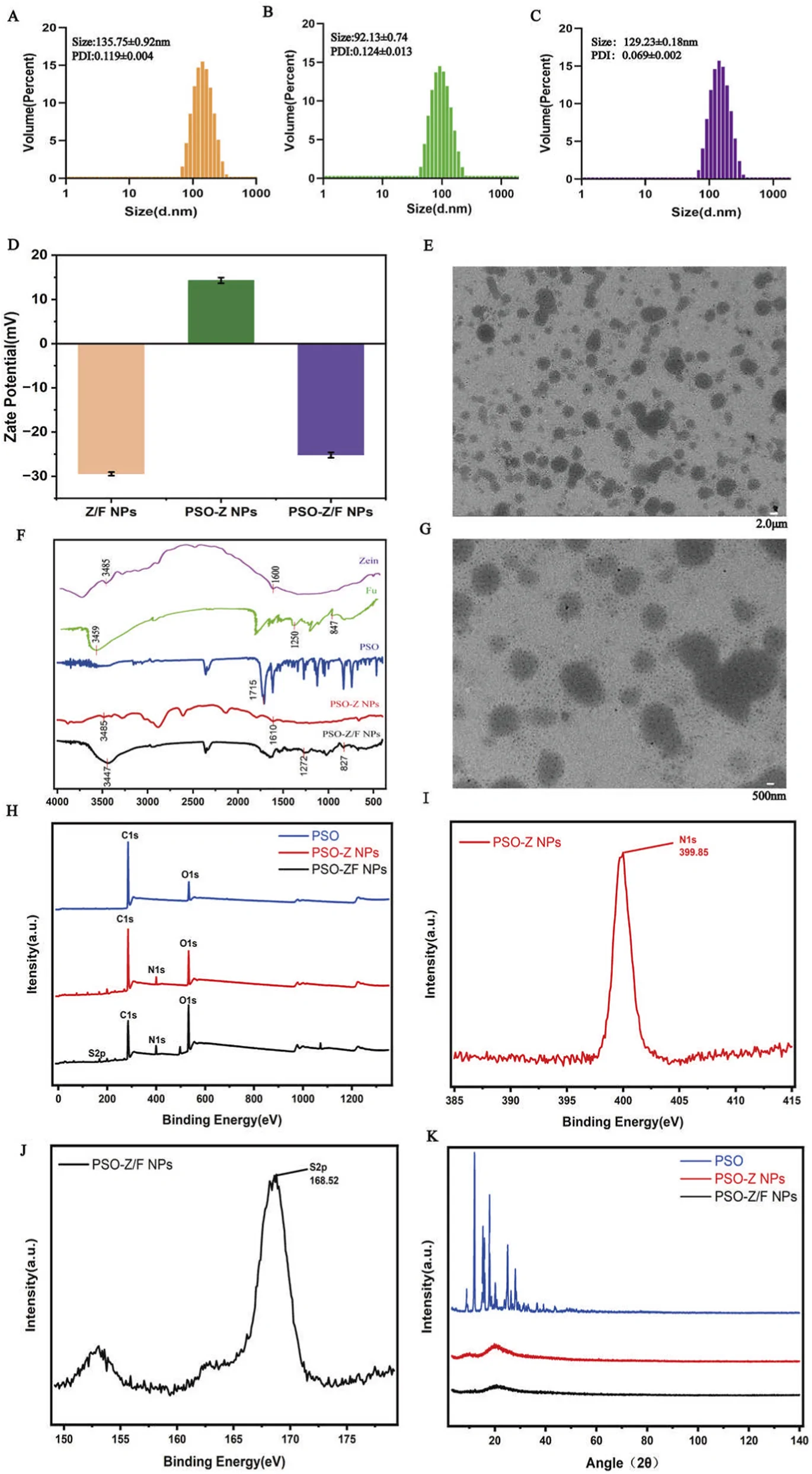
**(A–C)** Particle size of Z/F NPs,PSO-Z NPs and PSO-Z/F NPs; **(D)** ζ potential of Z/F NPs,PSO-Z NPs and PSO-Z/F NPs; TEM images of PSO-Z/F NPs **(E-G)**; **(F)** FTIR spectra of different samples; **(H–J)** XPS analysis, including survey spectrum and high-resolution spectra of N 1s and S 2p, and **(K)** XRD patterns.

**TABLE 1:** The load capacity and load efficiency of the optimally prepared PSO-Z/F NPs (n = 3).

PSO-Z/F NPs	1	2	3	Mean ± SD
EE (%)	73.92	72.03	73.43	73.13 ± 0.98
DL (%)	5.80	5.25	5.14	5.40 ± 0.35

#### 3.1.3 FTIR spectrum

Figure 1F shows the FTIR spectrum of the nanoparticles. Native zein displayed a characteristic absorption peak at 1,600 cm^-1^, which is indicative of its intrinsic secondary structure (Forato et al., 2003). Upon PSO encapsulation, this peak exhibited a redshift to 1,610 cm^-1^, a signature of β-sheet aggregate formation. This spectral shift confirms that PSO loading triggers conformational rearrangement of zein, involving the disruption of α-helical structures and the subsequent formation of β-sheet-rich aggregates. PSO revealed a characteristic peak at 1715 cm^-1^ corresponding to the C=O stretching of the α-pyrone ring, along with aromatic ring signals at 1,631 cm^-1^, and 1,575 cm^-1^ (Cui et al., 2018). However, this characteristic peak at 1715 cm^-1^ was absent in both PSO-Z NPs and PSO-Z/F NPs, because PSO was stably encapsulated within the hydrophobic microcavities formed by zein, where hydrophobic interactions and π–π stacking between the aromatic moiety of PSO and the hydrophobic amino acid residues of zein shielded the C=O functional group and reduced the intensity of its FTIR band. In addition, although the amide I band of native zein (∼1,600 cm^-1^) changed upon PSO encapsulation and fucoidan coating, these spectral changes cannot be directly assigned to specific secondary structure contents without deconvolution analysis, because the amide I band is a composite envelope of overlapping secondary structure contributions (Intze et al., 2025). Upon coating Fu onto the PSO-Z NPs, these peaks shifted to 1,272 cm^-1^ and 827 cm^-1^, respectively, suggesting hydrogen bonding and electrostatic interactions between the sulfated groups of Fu and the polar functional groups of zein. These results confirmed the successful formation of a stable Fu coating on the nanoparticle surface. Furthermore, the overlap of the O-H stretching peak of Fu with the N-H stretching peak of zein produced a broader and more intense absorption peak (3,447 cm^-1^), thereby further validating the interfacial interactions between zein and Fu (Liu et al., 2022).

#### 3.1.4 XPS characterization

The surface elemental composition was analyzed by X-ray photoelectron spectroscopy (XPS, Thermo SCIENTIFIC Nexsa, United States). Charge referencing was performed by aligning the adventitious C 1s peak to 284.8 eV for all samples. The uncertainty associated with charge referencing for insulating biopolymer samples was estimated to be approximately ±0.2 eV. Samples were pressed into conductive carbon tape and grounded to minimize differential charging. The surface elemental composition and intermolecular interactions of the samples were investigated (Figures 1H–J). Pure PSO exhibited characteristic binding energies of C 1s at 284.8 eV and O 1s at 532.62 eV. In PSO-Z NPs, an additional N 1s signal appeared at 399.85 eV, accompanied by a negative shift of O 1s to 532.0 eV. Following the incorporation of Fu, PSO-Z/F NPs demonstrated a further negative shift of O 1s to 531.63 eV, a variation in N 1s to 399.29 eV, and a distinct S 2p peak at 168.52 eV. An O 1s shift of this magnitude (0.5–1.5 eV) is well documented for hydrogen-bonded systems and supports the formation of intermolecular hydrogen bonds between the oxygen-containing functional groups of PSO and the biopolymer matrices (Beamson and Briggs, 1992). The negative N 1s shift of 0.56 eV lies within the range (0.4–0.7 eV) typically observed for electrostatic interactions between protonated amine groups and polyanions in polyelectrolyte complexe (Lawrie et al., 2007; Liu et al., 2022), and is thus consistent with strong electrostatic attractions between the positively charged zein and the negatively charged sulfate groups of Fu. XPS analysis provided further confirmation of the successful Fu coating. A distinct S 2p peak at 168.52 eV, characteristic of the sulfate groups in Fu, was observed in the spectrum of PSO-Z/F NPs but was absent from that of PSO-Z NPs, thereby verifying the presence of Fu on the nanoparticle surface. Taken together with the ζ potential reversal and the FTIR band shifts, these complementary supported the successful attachment of Fu onto the PSO-Z NPs. Collectively, these variations could be partially attributed to the synergistic interplay of hydrogen bonding, electrostatic attraction, and hydrophobic interactions during nanoparticle assembly and drug stabilization (Feng et al., 2025; Liu et al., 2022).

#### 3.1.5 XRD and DSC analysis

As presented in Figure 1K, XRD pattern of PSO exhibited numerous sharp peaks around 8°–44°(2θ), which demonstrated that PSO maintained a highly crystalline state (Matsushima et al., 1997; Momany et al., 2006). In contrast, no distinct characteristic peaks were observed in the pattern of PSO-Z/F NPs, indicating the amorphous nature of PSO-Z/F NPs. The DSC results (Figure 2A) shown that free PSO exhibited a distinct sharp endothermic melting peak at 135 °C–145 °C signifying its crystalline state. In contrast, zein displayed a typical endothermic peak associated with thermal denaturation within the range of 85 °C–120 °C, while Fu did not show a discernible melting peak, only exhibiting thermal events related to decomposition above 200 °C. Notably, the PSO-Z/F NPs lacked the characteristic crystalline melting peak of PSO at 135 °C–145 °C. The above results proved that PSO exists (Momany et al., 2006) in an amorphous or molecularly dispersed state within the Zein core, without undergoing recrystallization (Wang et al., 2022).

**FIGURE 2:**
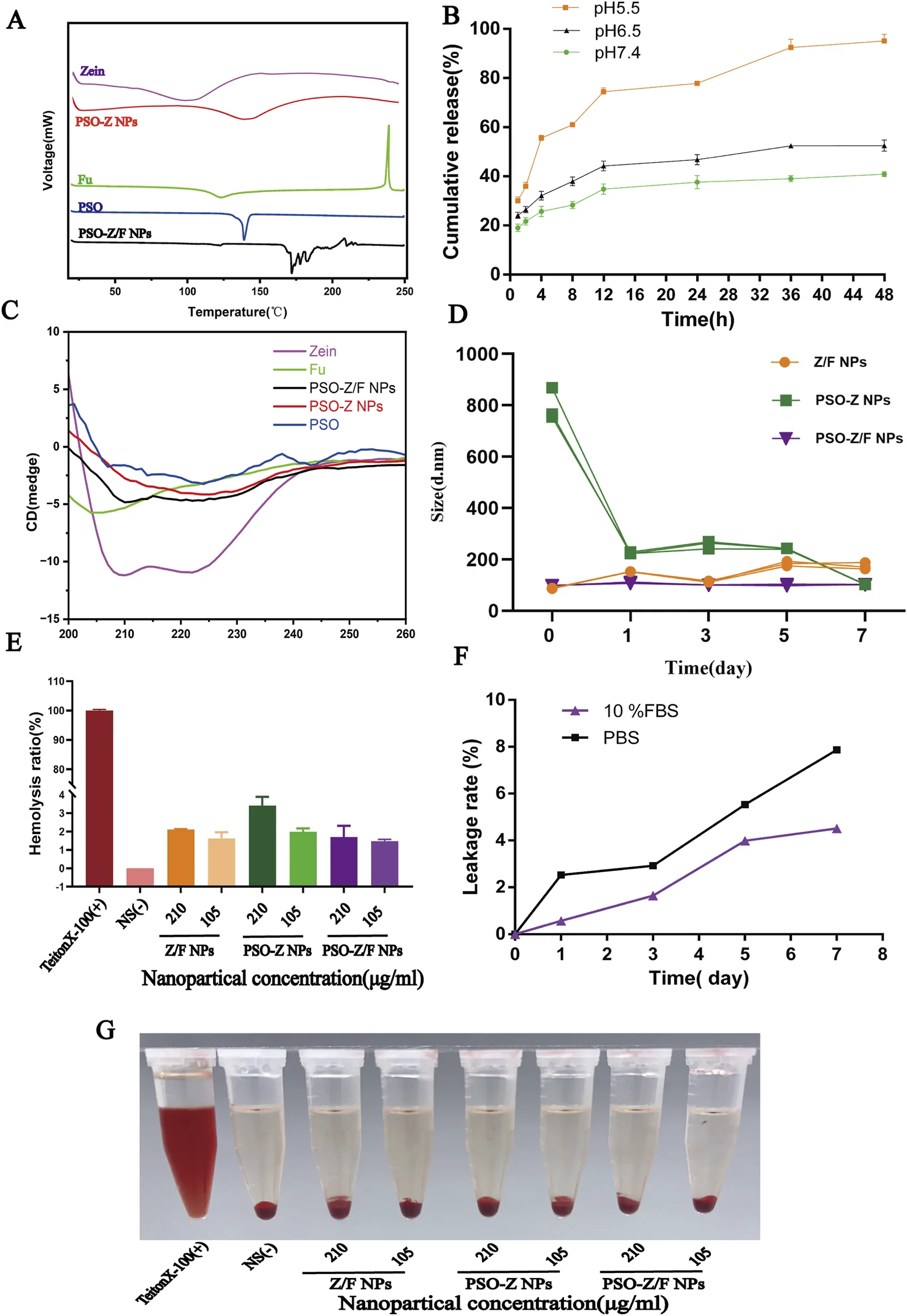
**(A)** DSC patterns; **(B)** Releasing profile of PSO-Z/F NPs under different conditions; **(C)** CD patterns; **(D)** Serum stability; **(E,G)** Hemolytic assay; **(F)** Nano-particle leakage rate.

#### 3.1.6 CD spectroscopy

The changes in the secondary structure of zein upon interaction with PSO were evaluated (Figure 2C). Zein displayed a characteristic CD spectrum dominated by α-helices, featuring two negative Cotton effects at 208 nm and 222 nm. This profile aligns with the typical CD characteristics of prolamin proteins that are rich in α-helical conformation (Forato et al., 2003; Yong et al., 2026). Upon PSO loading, these helical bands were substantially diminished, indicating a marked loss of α-helical content and a likely conformational shift toward β-sheet and random coil structures (Hong, 2014). Although light scattering by the nanoparticles may partially contribute to the observed signal attenuation, this conformational transition is corroborated by FTIR analysis. We therefore speculate that hydrophobic interactions and π–π stacking between PSO and Zein drive the formation of ordered, β-sheet-rich aggregates (Erickson et al., 2020; Newberry and Raines, 2019). Fu exhibited no detectable CD signal when measured alone, owing to its weak chiral absorption. However, upon being coated onto PSO-Z NPs, the complex showed a moderate increase in overall CD intensity, accompanied by further modulation of peak shape. This conformational change is driven by hydrogen bonding and electrostatic interactions between the sulfate groups of Fu and the peptide chain of the Zein, which collectively stabilize the overall nanocarrier conformation (El-Sheekh et al., 2025; Yong et al., 2026; Zhang et al., 2021). In contrast, PSO, being an achiral small molecule, did not exhibit any distinct CD signal independently. The absence of new characteristic peaks in the composite nanoparticles indicates that PSO is physically encapsulated within the hydrophobic core of zein, rather than being covalently conjugated or assembled into an ordered chiral microenvironment (Chen et al., 2026; Sah et al., 2006).

#### 3.1.7 Response release of nanoparticles

The pH-responsive drug release behavior renders it promising for intratumoral drug delivery, attributed to the acidic microenvironment of tumors. As depicted in Figure 2B, under pH values of 5.5 and 6.5, the cumulative release rate of PSO at 48 h were 92.4% ± 3.3% and 52.4% ± 1.0%, respectively, while only about 39.1% ± 1.3% at physiological conditions (pH 7.4). These results revealed that the PSO-Z/F NPs could achieve a pH-responsive drug release. This likely reflects the dual barrier formed by Zein and Fu, which degrade under acidic conditions as the negative charge on Fu declined and thus weakens its electrostatic interaction with Zein (Yong et al., 2026). Although independent solubility measurements were not performed, sink conditions were operationally maintained by withdrawing and replacing an equal volume of fresh medium at each sampling point, a widely adopted practice in nanoparticle release studies.

#### 3.1.8 *In vitro* stability investigation

As illustrated in Figure 2D, the stability of nanoparticles in serum was assessed for *in vivo* applications. For PSO-Z NPs, a dynamic size evolution was observed during incubation, characterized by an initial increase to approximately 800 nm followed by a gradual decrease. This behavior may be associated with the elevated surface charge of the uncoated Zein nanoparticles. The high surface charge facilitates the rapid adsorption of serum proteins, leading to their transient aggregation during the initial stage (Tenzer et al., 2013). With prolonged incubation, a stable protein canopy developed gradually, leading to structural rearrangement on the nanoparticles’ surface. Consequently, the particle size decreased without experiencing irreversible aggregation or precipitation (Malhotra et al., 2024). In contrast, the particle size of PSO-Z/F NPs remained stable throughout the incubation period, with no significant fluctuations. This stability indicates that the hydrophilic fucoidan shell effectively screens surface charges, suppresses non-specific protein adsorption, and prevents transient aggregation, thereby conferring robust colloidal stability in biological environments (Zhang et al., 2021). The results of *in vitro* serum stability tests indicated that the Fu coating layer significantly contributes to the structural stability of nanoparticles under physiological conditions, suggesting its potential to prolong *in vivo* circulation time. However, this inference requires validation through subsequent pharmacokinetic or circulatory experiments. Figure 2F shown the drug leakage profile of PSO-Z/F NPs. The results showed that the cumulative drug leakage rate remained below 10% within 7 days under both storage conditions, suggesting the exceptional structural stability of PSO-Z/F NPs. This quality enables them to efficiently preserve PSO and prevent premature drug release in physiological settings, ensuring the reliable delivery of PSO to the designated tumor site. These findings align with the stability attributes of natural polysaccharides and protein-based biopolymers documented in earlier research studies (Dumontel et al., 2023; Trofymchuk et al., 2019).

#### 3.1.9 Hemolysis experiments

Hemolysis experiments shown that in the positive control group (0.1% Triton X-100 solution) a clear red color was observed, indicating red blood cell membrane damage and hemolysis. Conversely, the supernatants from the negative control group, Z/F NPs, PSO-Z NPs, and PSO-Z/F NPs groups were colorless and transparent, without hemolysis or condensation (Figure 2E). The hemolysis rates of Z/F NPs, PSO-Z NPs, and PSO-Z/F NPs were all less than 3% (Figure 2G), suggesting minimal hemolytic activity and favorable biological safety profiles (Lan et al., 2026; Li et al., 2025).

### 3.2 *In vitro* cytotoxicity and cellular uptake

#### 3.2.1 *In vitro* cytotoxicity

A CCK-8 toxicity assay was conducted to evaluate the cytotoxic effects of PSO-Z/F NPs on 4T1 cells before their intended biological application. Figure 3A illustrates a consistent increase in cytotoxicity among the treatment groups. Compared with the PSO group, the PSO-Z NPs group exhibited significantly greater cytotoxicity. After surface modification with Fu, the PSO-Z/F NPs group showed a further increase in cytotoxicity, indicating that the Fu coating increased tumor binding and promoted tumoricidal activity (Hsu et al., 2020). These enhancements could be attributed to the inherent tumor-targeting properties of zein and Fu, potentially facilitating increased cellular uptake of the nanoparticles by tumor cells. Additionally, Z/F NPs demonstrated negligible cytotoxicity.

**FIGURE 3:**
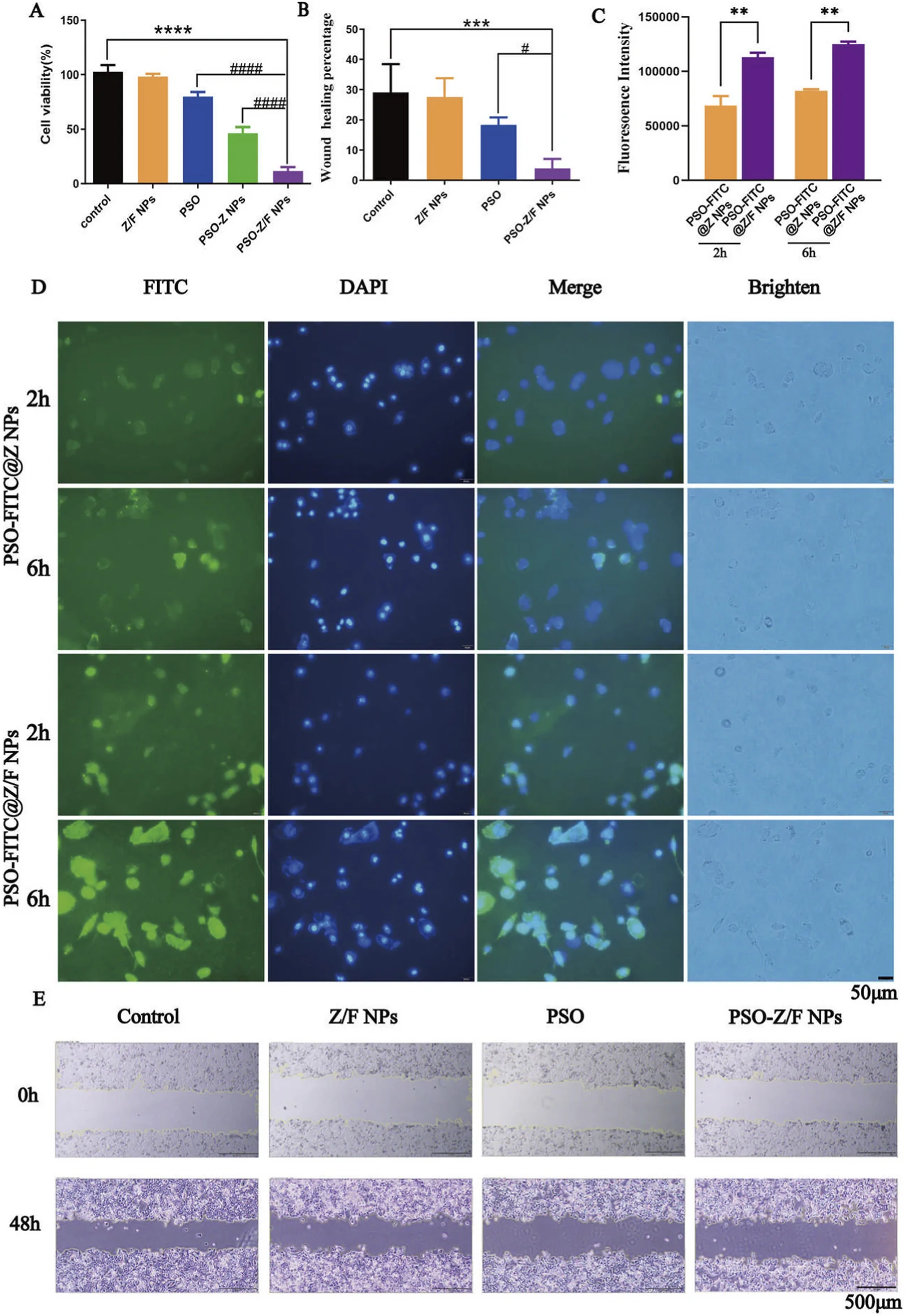
**(A)** Cell viability of 4T1 cells assessed using CCK-8 after various treatments. **(B)** 4T1 cell wound healing rate observed over 48 h after different treatments. **(C)** Flow cytometric quantification; **(D)** Uptake of PSO-FITC@Z NPs and PSO-FITC@Z/F NPs by 4T1 cells at various incubation durations. **(E)** Visual representation of wound closure at 0 and 48 h. “#” denotes statistical significance between groups. ***, *P* < 0.001 compared to control; #, *P* < 0.05; ###, *P* < 0.001, ####, *P* < 0.0001.

#### 3.2.2 Cell uptake experiments

To investigate the cellular uptake efficiency of the PSO-Z/F NPs, the nanoparticles were labeled with FITC and analyzed using FM and FCM. As shown in Figure 3C, 2 h post-administration, fluorescence signals were observed in both PSO-FITC@Z NPs and PSO-FITC@Z/F NPs groups. Notably, the fluorescence intensity in PSO-FITC@Z/F NPs group was 1.6-fold higher than in PSO-FITC@Z NPs group, suggesting a higher internalization efficiency (Figure 3D). This could be ascribed to the improved specific tumor-targeting ability of nanoparticles mediated by Fu coating, which in turn facilitates drug internalization (Chiang et al., 2021; Lin et al., 2024).

### 3.3 Wound healing, cell migration and invasion assay

Wound healing and transwell assays were employed to assess the inhibitory effect of PSO-Z/F NPs on cell metastasis *in vitro*. Quantitative analysis of the scratch wound images (Figures 3B,E; Supplementary Table S3) showed that the corresponding grayscale-derived wound healing rates of 4T1 cells within 48 h were 29.13% ± 3.33% (control group), 18.34% ± 2.52% (PSO), and 5.02% ± 2.86% (PSO-Z/F NPs). Compared with the control and PSO groups, the wound closure rate in the PSO-Z/F NPs group decreased significantly (*P* < 0.001 and *P* < 0.05, respectively).

Based on wound healing experiment, migration and invasion assays were performed to investigate how PSO-Z/F NPs affect longitudinal mobility of 4T1 cells (Figures 4A,B,D) and we found that PBS and Z/F NPs penetrated much (in fact covering nearly the entire lower surface) suggesting highly metastatic 4T1 cells. However, compared with the PSO group, the numbers of migrated and invaded cells were significantly reduced in the PSO-Z/F NPs group. Specifically, the number of migrated cells decreased from 220 ± 33 to 146 ± 20 (*P* < 0.01), while the number of invaded cells decreased from 144 ± 21 to 90 ± 8 (*P* < 0.05). Collectively, these findings suggest that PSO-Z/F NPs may suppress the migratory and invasive capacities of 4T1 cells *in vitro.*

**FIGURE 4:**
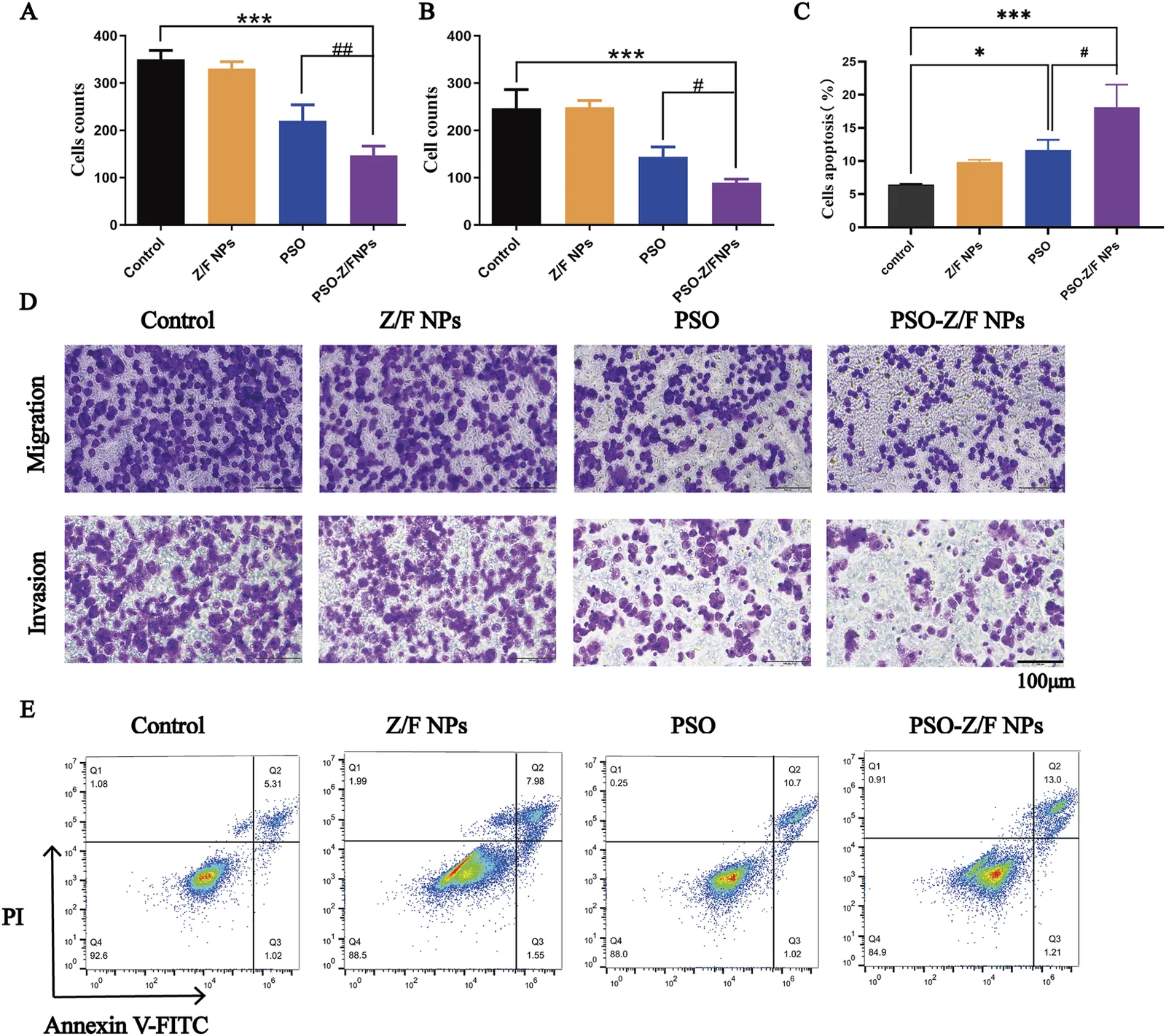
**(A)** Changes in migration rate and **(B)** invasion rate of 4T1 cells treated with different formulations; **(C-E)** Apoptosis induction in 4T1 cells by distinct formulations assessed *via* flow cytometry; **(D)** Microscopic visualization of 4T1 cell migration and invasion after different treatments on transwell membranes at 48 h. “#” denotes statistical significance between groups. *, *P* < 0.05; **, *P* < 0.01; ***, *P* < 0.001 compared to control; #, *P* < 0.05; ##, *P* < 0.01.

### 3.4 Apoptosis experiments

Following the confirmation of the anti-migration and anti-invasion effects of PSO-Z/F NPs, apoptosis rates induced by various treatments using Annexin V-FITC/PI staining were assessed. The percentages of viable, early apoptotic, late apoptotic, and necrotic cells were shown in Supplementary Table S4. As revealed in Figures 4C,E and Supplementary Table S4, the Z/F NPs group showed an apoptotic rate of 9.86% (1.45% ± 0.11% early and 8.41% ± 0.37% late apoptosis), which was not significantly different from that of the control group (6.45%; 0.73% ± 0.26% early and 5.72% ± 0.36% late apoptosis). In contrast, treatment with PSO-Z/F NPs markedly increased the apoptotic rate to 17.98% (*P* < 0.01), including 1.15% ± 0.28% early apoptotic cells and 16.83% ± 3.33% late apoptotic cells. Notably, this rate was significantly higher than that in the free PSO group (*P* < 0.05), possibly owing to the more efficient cellular internalization of the nanoparticles by 4T1 cells. These results further suggest that the nanoparticle formulation may offer potential advantages over the free drug.

### 3.5 *In vivo* biodistribution

Based on the significant anticancer efficacy of PSO-Z/F NPs, *in vitro*, we further investigated the *in vivo* biodistribution. As revealed in Figure 5, intense fluorescence signals were observed in both PSO-cy5.5@Z NPs and PSO-cy5.5@Z/F NPs groups post-administration, predominantly localized in the tumor, liver, and lung. For the PSO-cy5.5@Z/F NPs group, the fluorescence intensity progressively rose and peaked at 24 h, then gradually decreased, persisting notably even at 96 h in the tumor area. Additionally, the PSO-cy5.5@Z NPs group displayed a similar trend but with markedly lower fluorescence levels across all time points, peaking at 12 h. The relatively strong fluorescence signals in the liver may be associated with uptake by the mononuclear phagocyte system, which is commonly involved in the recognition and clearance of intravenously administered nanoparticles (Blanco et al., 2015; Moghimi et al., 2001; Owens and Peppas, 2006). The lung signals may reflect nanoparticle distribution and/or retention in the pulmonary microvasculature, which can be influenced by nanoparticle physicochemical properties, including particle size and surface characteristics (Albanese et al., 2012; De Jong et al., 2008; Longmire et al., 2008). These potential mechanisms were not directly investigated and require further study. Collectively, the findings indicate that PSO-Z/F NPs exhibited higher and more prolonged tumor-associated fluorescence signals than PSO-Z NPs, suggesting enhanced accumulation and retention in tumor tissue in the current experimental setup. Nevertheless, these outcomes alone do not confirm active tumor targeting or extended systemic circulation, necessitating additional pharmacokinetic and mechanistic studies.

**FIGURE 5:**
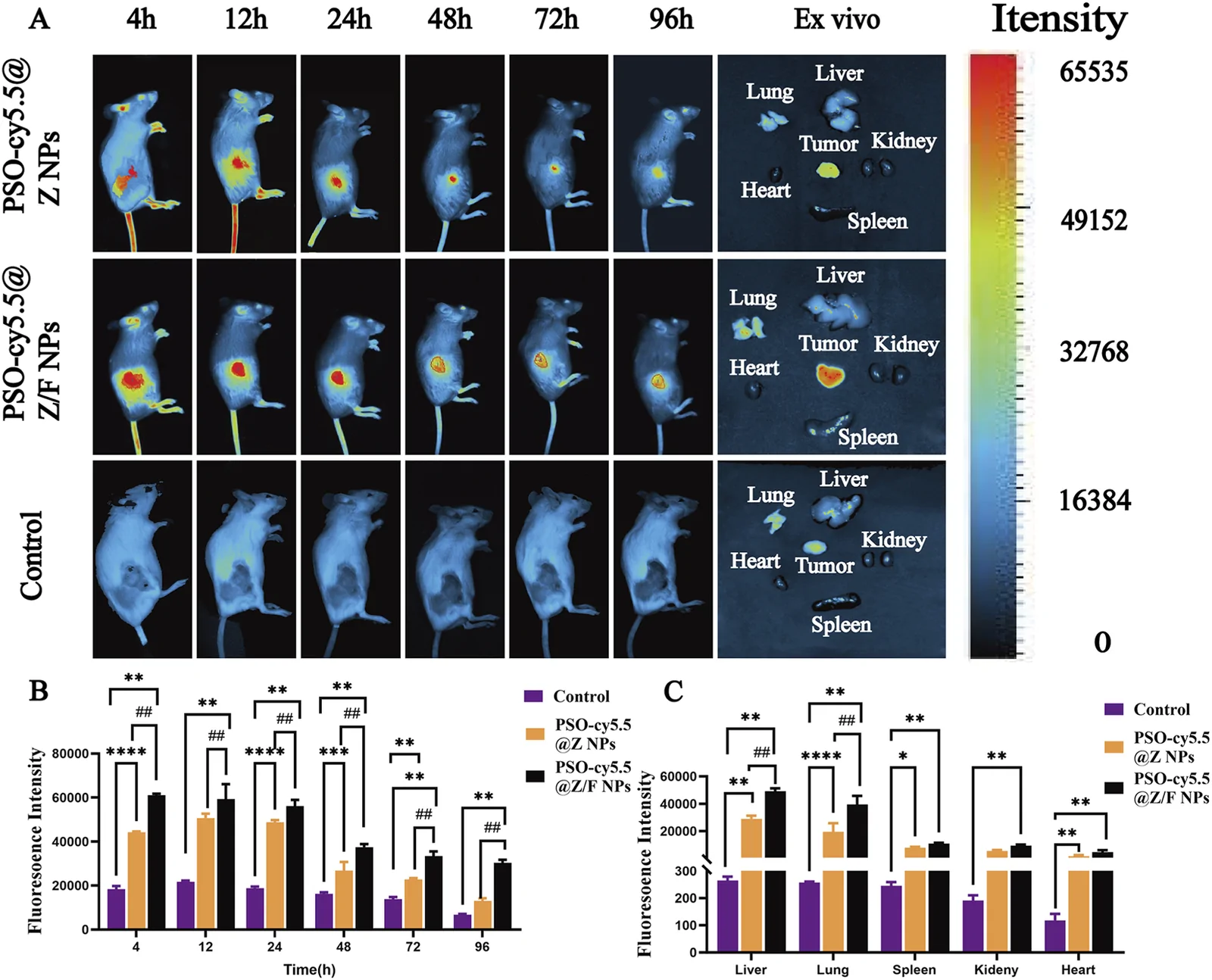
**(A)** Fluorescence pictures of mice with 4T1 tumors (n = 3) injected with PSO-cy5.5@Z NPs and PSO-cy5.5@Z/F NPs shown *in vivo* and post-mortem at different time intervals. **(B)** Semi-quantitative evaluation of PSO-cy5.5@Z NPs and PSO-cy5.5@Z/F NPs buildup in tumors. **(C)** NPs distribution in various organs evaluated utilizing fluorescence intensity analysis software. “#” denotes statistical significance between groups. *, *P* < 0.05; **, *P* < 0.01; ***, *P* < 0.001, compared to control; #, *P* < 0.05; ##, *P* < 0.01.

### 3.6 *In vivo* antitumor efficacy

Encouraged by the *in vivo* biodistribution findings, we next evaluated the antitumor efficacy of PSO-Z/F NPs in a unilateral 4T1 tumor-bearing mouse model. The procedures for establishing the tumor model and administering treatment are shown in Figure 6A. As depicted in Figures 6D,E, the growth of the tumor in the PSO-Z/F NPs group was significantly inhibited compared to the PBS control group (*P* < 0.05). Moreover, the inhibitory effect on tumor growth in the PSO-Z/F NPs group was markedly greater than that observed in the free PSO group (*P* < 0.05). This could be attributed to the poor tumor enrichment ability of free PSO and its rapid clearance from the bloodstream. In contrast, the tumor growth in the Z/F NPs group did not exhibit significant changes compared to the PBS group, suggesting that the blank nanocarrier did not exhibit apparent antitumor activity under the present experimental conditions.

**FIGURE 6:**
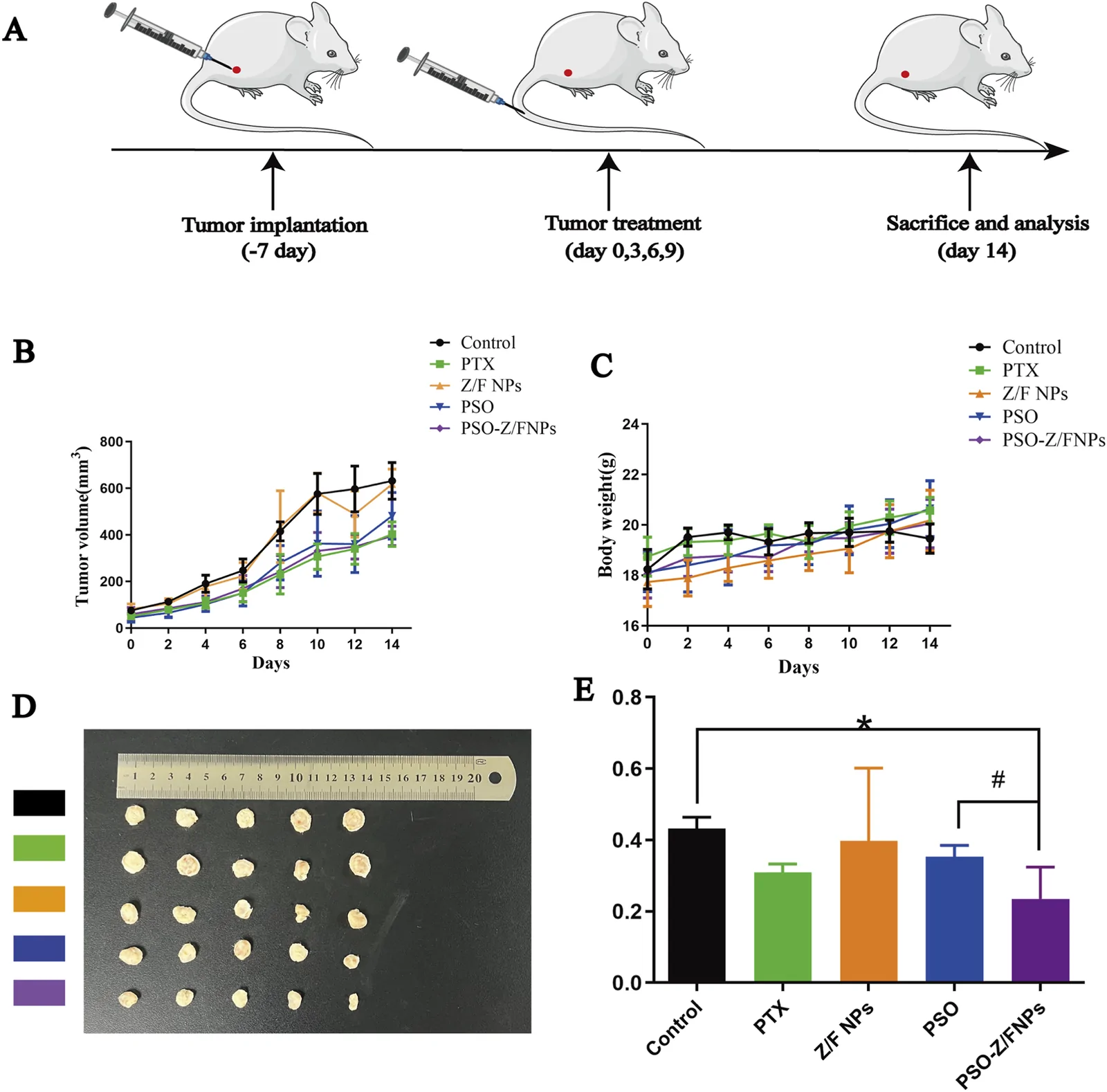
**(A)** Establishment of mouse model with 4T1 tumors and treatment strategy diagram. **(B)** Tumor volume comparison among treatment groups (n = 5). **(C)** Body weight fluctuation graph (n = 5). **(D)** Day 20 tumor images from various treatment groups (n = 5). **(E)** Tumor weight outcomes across treatment cohorts (n = 5). “#” denotes statistical significance between groups. *, *P* < 0.05 compared to control. #, *P* < 0.05.

Consistently, mice treated with PSO-Z/F NPs showed the smallest tumor volumes and tumor weights among all treatment groups (Figures 6B,E). These results indicate that PSO-Z/F NPs exhibit improved *in vivo* antitumor efficacy compared to free PSO. However, the current data do not directly establish whether this enhanced efficacy is linked to targeted tumor delivery, increased cellular uptake, or prolonged *in vivo* drug release. Further studies are necessary to elucidate the mechanisms underlying the improved therapeutic effect. Additionally, no significant body weight loss occurred during treatment, suggesting that the nanoparticles demonstrate acceptable *in vivo* tolerability (Figure 6C).

### 3.7 Biosafety analysis *in vivo*

As shown in Figure 7B H&E staining was used for qualitative histopathological evaluation of the major organs, including the heart, liver, spleen, lung, and kidney. No apparent treatment-related histopathological abnormalities, such as inflammatory cell infiltration, hemorrhage, necrosis, cellular degeneration, or disruption of tissue architecture, were observed in any treatment group. Subsequent TUNEL staining evaluated apoptosis qualitatively in tumor tissues (Figure 7A). Green fluorescent signals, indicating apoptotic cells, were prominently present in tumor sections from the PSO-Z/F NPs group, while no TUNEL-positive signals were evident in the control group. The PSO-Z/F NPs group exhibited more pronounced TUNEL-positive signals than the PSO group, indicating increased tumor cell apoptosis post PSO-Z/F NPs treatment.

**FIGURE 7:**
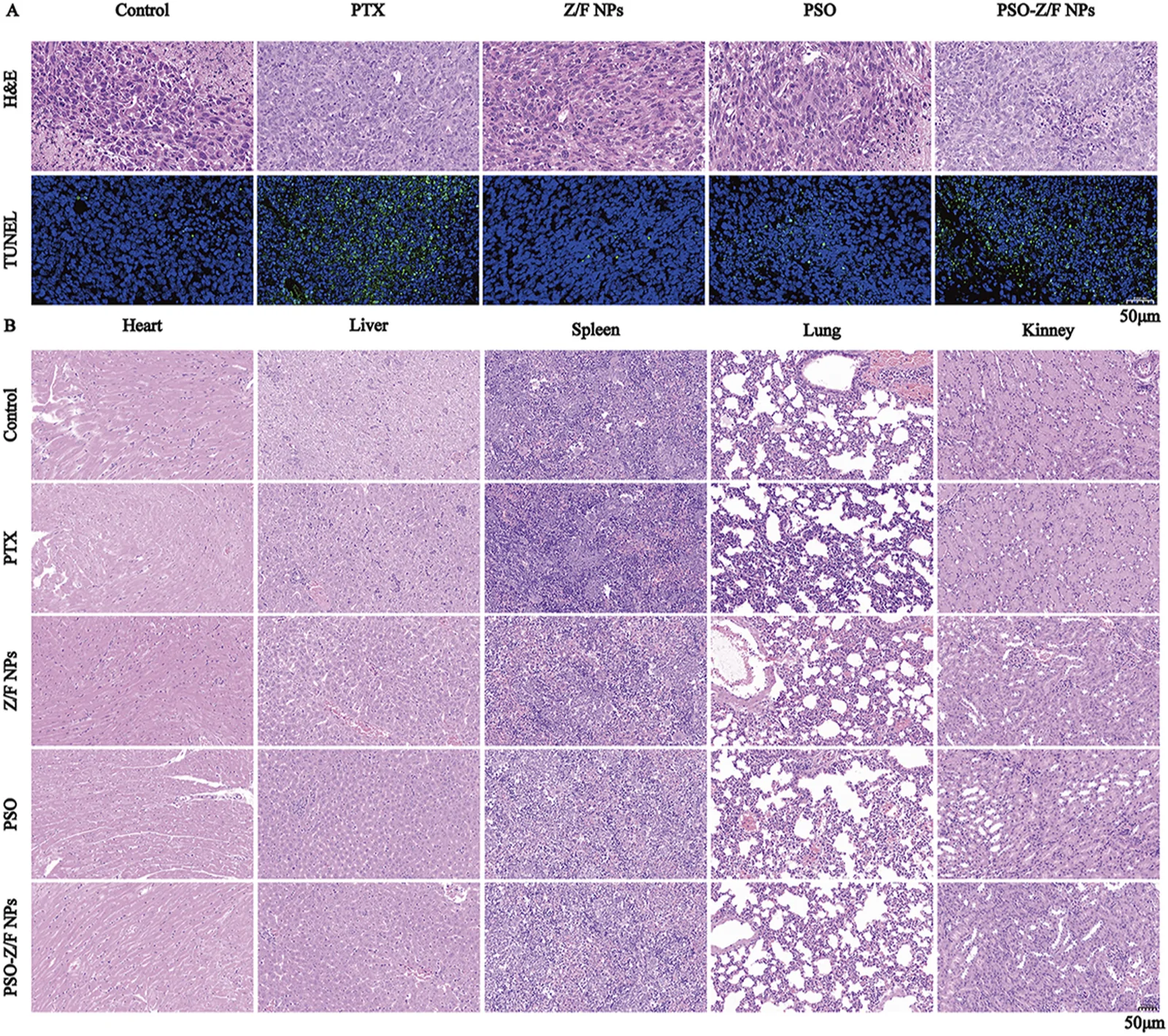
**(A)** H&E staining of mice major organs and tumors from different treatment groups. **(B)** TUNELL staining of tumors from different treatment groups. The images were captured at 10× objective. (n = 5).

## 4 Conclusion

In this study, zein and fucoidan, both of which exhibit natural bioactivity, were utilized as nanocarriers for the delivery of psoralen in breast cancer treatment. Spectral analysis suggests that PSO induces conformational rearrangement of Zein, characterized by partial α-helix unwinding and unfolding of the protein chain. This reorganization is likely to create a larger hydrophobic region that facilitates drug accommodation. Concurrently, these conformational changes may strengthen the interfacial interactions between Zein and Fu by promoting additional hydrogen bonding, electrostatic contacts, and hydrophobic association. Collectively, these concerted interactions may drive the formation of a more compact nanoparticle core, thereby enhancing overall structural stability. The fabricated PSO-Z/F NPs featured nanoscale dimensions with a low PDI, pH-sensitive property, sustained release behavior, low drug leakage rate and hemolysis rate. These physicochemical properties confer exceptional tumor-targeting capabilities upon it. *In vitro* experiments suggested that the potent cytotoxicity of this nanoparticle, leading to a significant induction of apoptosis in 4T1 breast cancer cells and the inhibition of their invasion. *In vivo* evaluations indicated that PSO-Z/F NPs demonstrate acceptable preliminary biocompatibility under the tested conditions. No apparent treatment-related histopathological abnormalities were observed in the major organs based on blinded histopathological evaluation, and no significant body weight loss occurred during treatment, suggesting acceptable preliminary *in vivo* tolerability under the tested conditions. The formulation is also linked to enhanced drug accumulation at tumor sites, which likely contributed to the observed inhibition of tumor growth. Overall, the sulfated polysaccharide-coated protein nanoparticles developed in this study offer a promising delivery method for enhancing the therapeutic application of psoralen in breast cancer. Nevertheless, further studies are needed to comprehensively investigate their systemic circulation profile, long-term safety, antitumor mechanisms, and therapeutic efficacy in metastasis model. In addition, a few limitations remain, primarily the lack of comprehensive safety evaluations and the use of incomplete control groups. Therefore, future studies should incorporate comprehensive safety assessments and appropriate control groups to further validate the biosafety profile and clarify the contribution of the fucoidan coating.

## Data Availability

The original contributions presented in the study are included in the article/Supplementary Material, further inquiries can be directed to the corresponding authors.
