# Supplementary material for: Sulfated polysaccharide-coated protein nanoparticles for enhanced delivery of psoralen in breast cancer therapy
Source: Front Pharmacol. 2026 Oct 20;17:1888270. doi: 10.3389/fphar.2026.1888270 (PMC13727547; doi:10.3389/fphar.2026.1888270)
Supplement: Supplementary file 1 [file DataSheet1.pdf]

Supplementary information

Sulfated polysaccharide-Coated Protein Nanoparticles for Enhanced Delivery of Psoralen in Breast Cancer Therapy

Huiqiong Guo[a,1], Jing Liang[a,1], Haili Huang[b], Zimo Zhang[b], Xin Huang[a], Zongxi Sun[b,*],Yinghong Huang[a,c,**]

[a] Zhuang Medicine Clinical Medical College, Guangxi University of Chinese Medicine, Nanning 530201, China

[b] The second Clinical Medical School, Guangxi University of Chinese Medicine, Nanning, 530011, China

[c] School of Chinese Materia Medica, Beijing University of Chinese Medicine, Beijing 100105, China

E-**mail addresses:** sunzx@gxtcmu.edu.cn (Zongxi Sun), gzyhyh2026@163.com (Yinghong Huang).

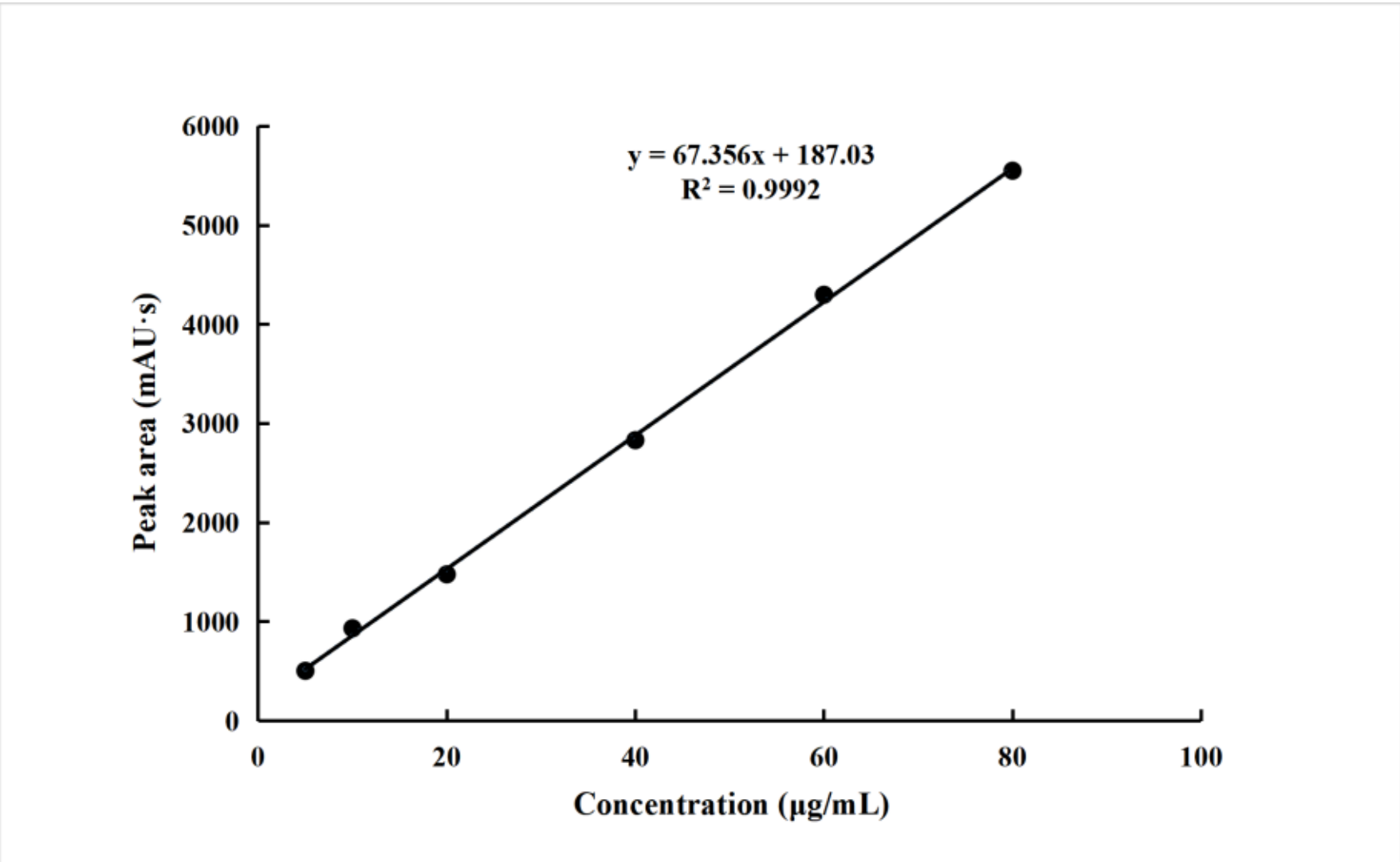

Figure S1. Calibration curve of PSO

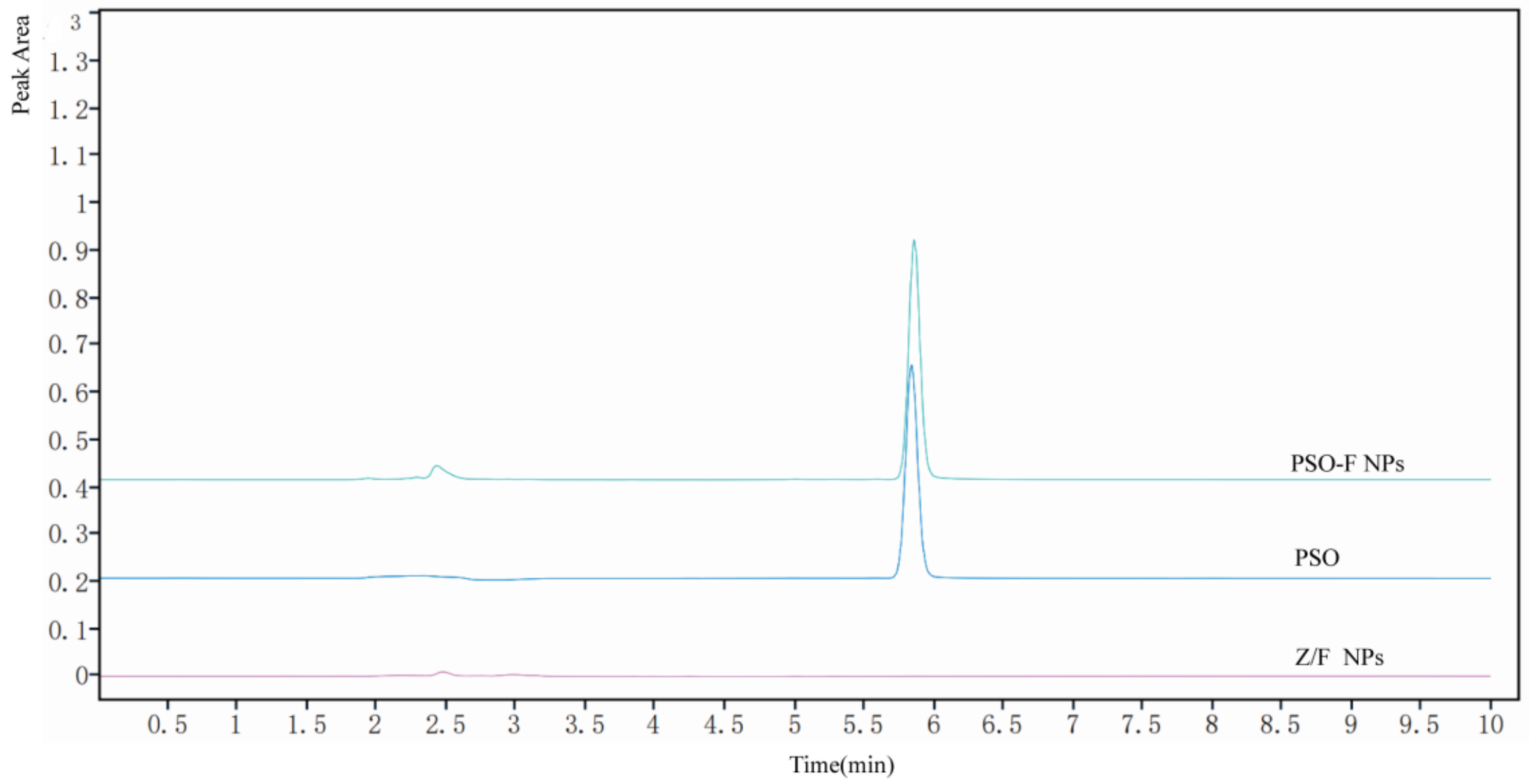

Figure S2. Calibration curve of PSO

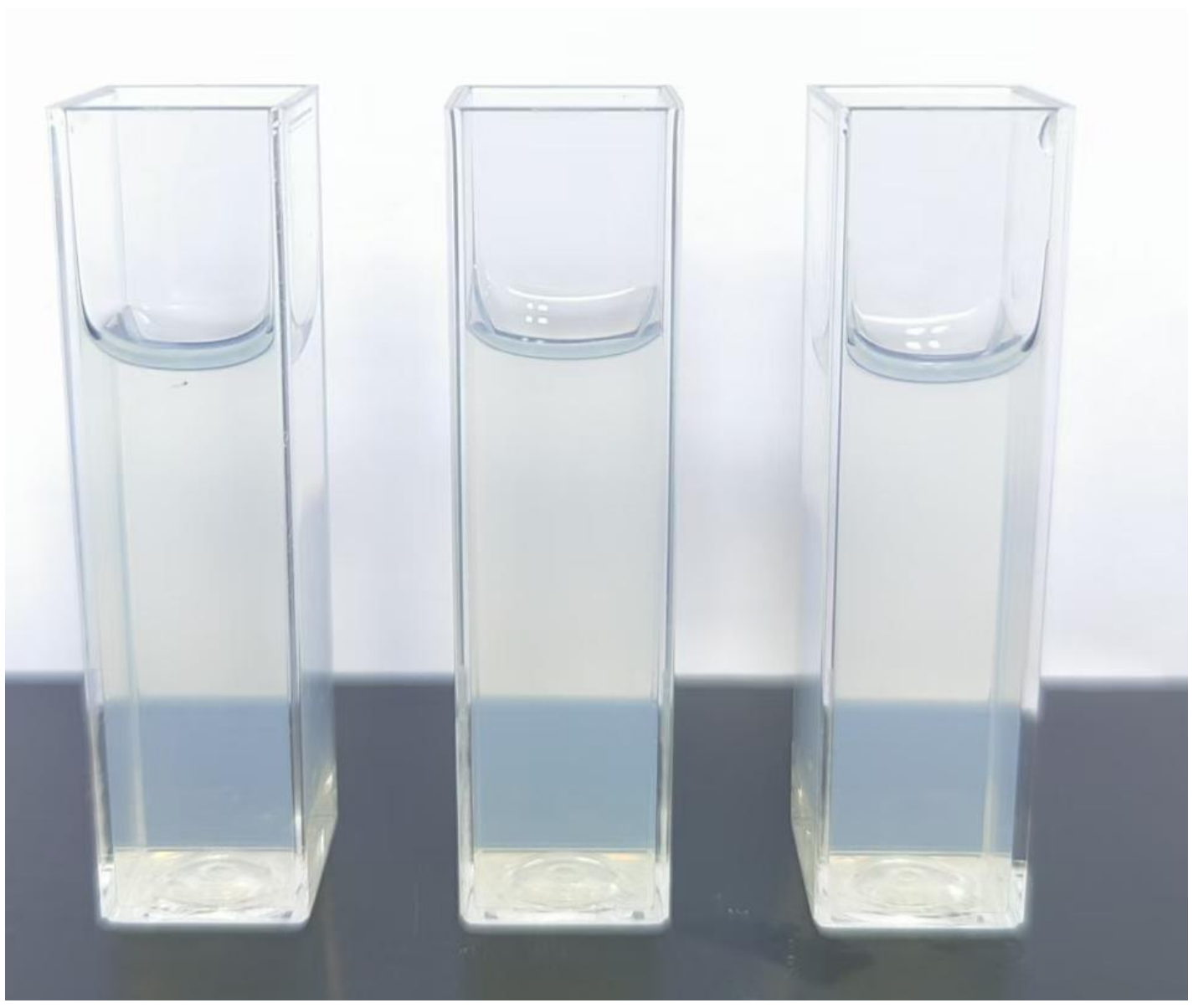

Fig. S3. Appearance of PSO-Z/F NPs

Table S1 Effect of different zein concentrations on nanoparticle properties. (n = 3)

zein（mg/mL）	60	70	80
Size (nm)	113 ± 0.92	128 ± 1.21	139 ± 1.34
PDI	0.167 ± 0.021	0.169 ± 0.019	0.187 ± 0.033

Table S2 Effects of different PSO:zein ratios on the properties of nanoparticles. (n = 3)

PSO:zein ratios (w/w)	1:10	1:20	1:30
Size (nm)	92 ± 1.54	104 ± 1.37	131 ± 0.91
PDI	0.111 ± 0.012	0.149± 0.009	0.102 ± 0.015
EE(%)	66.752 ± 1.24	73.951 ± 1.31	68.444± 0.99

Table S3 Mean gray value of the wound region at different time points (n = 4)

Group	Gray value at 0h (a.u.)	Gray value at 48h (a.u.)	grayscale-derived wound healing rate (%)
Control	131701.659 ± 5054.106	91943.320 ± 3221.388	29.13 ± 3.33
Z/F NPs	150068.625 ± 5822.026	106037.265 ± 3318.061	27.61 ± 6.18
PSO	145672.943 ± 4550.242	118933.943 ± 3719.967	18.34 ± 2.52
PSO-Z/F NPs	134604.941 ± 4043.314	129027.579 ± 3788.725	5.02 ± 2.86

Table S4 Annexin V/PI flow cytometric analysis of cell apoptosis (n = 3)

Group	Viable cells (%)	Early apoptotic cells (%)	Late apoptotic cells (%)	Necrotic cells (%)
Control	92.47 ± 0.12	0.73 ± 0.26	5.72 ± 0.36	1.09 ± 0.07
Z/F NPs	87.97 ± 0.47	1.45 ± 0.11	8.41 ± 0.37	2.17 ± 0.16
PSO	88.03 ± 1.45	1.29 ± 0.14	10.50 ± 1.31	0.28 ± 0.10
PSO-Z/F NPs	80.87 ± 3.52	1.15 ± 0.28	16.83 ± 3.33	1.05 ± 0.15
